# Molecular Networks of Redox Dysregulation in Fetal Alcohol Spectrum Disorders: Mechanisms and Therapeutic Prospects

**DOI:** 10.3390/antiox15040470

**Published:** 2026-04-10

**Authors:** Xiaoqing Wang, Shao-Yu Chen

**Affiliations:** 1 Department of Pharmacology and Toxicology, Health Sciences Center, University of Louisville, Louisville, KY 40292, USA; xiaoqing.wang@louisville.edu; 2 Alcohol Research Center, University of Louisville, Louisville, KY 40292, USA

**Keywords:** fetal alcohol spectrum disorders, redox dysregulation, reactive oxygen species, antioxidants

## Abstract

Fetal alcohol spectrum disorders (FASD) encompass a continuum of developmental abnormalities caused by prenatal alcohol exposure, resulting in persistent neurodevelopmental and structural defects. Accumulating evidence indicates that redox dysregulation plays a central role in the pathogenesis of FASD. Ethanol disrupts cellular redox homeostasis by promoting excessive reactive oxygen species production and depleting endogenous antioxidants, thereby perturbing key redox-sensitive molecular networks. Dysregulation of these pathways leads to mitochondrial dysfunction, endoplasmic reticulum stress, lysosome dysfunction, and disrupted cellular processes, including proliferation, differentiation, and migration, while also promoting apoptosis and neuroinflammation, ultimately leading to the developmental abnormalities characteristic of FASD. Recent studies demonstrate that antioxidant supplementation or targeted modulation of redox-sensitive signaling can mitigate these deleterious effects in preclinical models. This review synthesizes current knowledge of the molecular networks underlying redox dysregulation in FASD and discusses emerging antioxidant and dietary interventions with therapeutic potential. Elucidating these mechanisms provides critical insight into the pathogenesis of FASD and may inform the development of effective strategies for the prevention and treatment of FASD.

## 1. Introduction

Fetal alcohol spectrum disorders (FASD) refer to a range of lifelong physical, cognitive, and behavioral impairments resulting from prenatal alcohol exposure [1,2,3,4]. FASD encompasses several diagnostic categories, including fetal alcohol syndrome (FAS), alcohol-related neurodevelopmental disorder (ARND), alcohol-related birth defects (ARBD), and neurobehavioral disorder associated with prenatal alcohol exposure (ND-PAE) [1,2,5]. Among these, FAS is the most well-characterized and severe manifestation. It is defined by growth deficiencies, a distinct pattern of facial dysmorphology, including short palpebral fissures, a smooth philtrum, and a thin upper lip, and structural and/or functional abnormalities of the central nervous system [5,6]. In contrast, ARND, ARBD, and ND-PAE lack the distinctive facial features of FAS. ARND is characterized primarily by neurodevelopmental and behavioral impairments associated with prenatal alcohol exposure, whereas ARBD involves structural congenital abnormalities affecting organs such as the heart, kidney, or skeleton. ND-PAE is defined primarily by deficits in cognition, self-regulation, and adaptive functioning [7,8]. Despite these diagnostic distinctions, these subtypes are closely related and frequently co-occur, reflecting the complex and multifactorial teratogenic effects of prenatal alcohol exposure on developing fetal tissues and organs.

FASD represents a major global public health concern due to its high prevalence, lifelong consequences, and substantial economic burden [1,9]. Epidemiological estimates indicate that the global prevalence of FASD among children and youth is approximately 7.7 per 1000, and that approximately 1 in 13 pregnant women who consumed alcohol during pregnancy gives birth to a child with FASD [9]. Prevalence rates are markedly higher in certain regions, including Eastern Europe, South Africa, and indigenous populations in North America and Australia. However, the true prevalence of FASD is likely underestimated owing to diagnostic challenges, underreporting, and variability in surveillance systems. FASD imposes a substantial burden on affected individuals, families, and healthcare and social support systems [10,11]. For example, the annual societal cost of FASD in the United States has been estimated to be over $200 billion [12]. Collectively, these underscore the critical need to elucidate the mechanism underlying FASD pathogenesis to inform the development of effective preventive and therapeutic strategies that can mitigate both clinical outcomes and societal costs.

Accumulating evidence indicates that the adverse effects of alcohol on developing embryos are largely mediated by oxidative stress, with reactive oxygen species (ROS) emerging as a central contributor in the pathogenesis of FASD [13,14,15,16,17]. Embryonic tissues are uniquely vulnerable to such redox imbalance because they possess limited antioxidant capacity while relying on precisely regulated ROS-dependent signaling to control cell fate, proliferation, and morphogenesis; thus, even modest perturbation can cause cellular damage and embryonic development [18,19]. Seminal studies by Kotch et al. [16] demonstrated that free radical–mediated oxidative damage is a major mechanism underlying ethanol-induced teratogenesis, providing one of the earliest experimental links between ethanol and embryonic oxidative injury [16]. Subsequent investigations have expanded upon this concept by demonstrating that ethanol exposure elevates ROS levels and induces oxidative damage to lipids, proteins, and DNA of developing fetal tissues, including the brain, which is particularly vulnerable to oxidative insult due to its high lipid content and the high metabolic rate [15,20]. In addition, oxidative stress results in mitochondrial dysfunction in embryonic tissues [1,21], triggers inflammatory responses, and disrupts normal cellular processes, including cell proliferation, differentiation, migration, and survival through redox dysregulation of interconnected molecular networks. Together, these mechanisms contribute to the structural and functional abnormalities characteristic of FASD.

This article reviews and integrates current understanding of the molecular networks of redox dysregulation underlying the pathogenesis of FASD and highlights emerging therapeutic strategies targeting these mechanisms for the prevention and intervention of FASD. We delineate how prenatal alcohol exposure induces excessive ROS production and depletes endogenous antioxidants, thereby disrupting key redox-regulatory pathways. The review also describes how the dysregulation of these interconnected networks impairs mitochondrial functions, induces endoplasmic reticulum stress and lysosome dysfunction, which in turn impairs cell proliferation, differentiation, and migration, and induces apoptosis and neuroinflammation, ultimately leading to the developmental abnormalities characteristic of FASD. In parallel, this review synthesizes current experimental and translational evidence supporting antioxidant-based interventions and modulation of redox-sensitive signaling as potential strategies to restore redox homeostasis and mitigate FASD-related outcomes. By integrating mechanistic insights with therapeutic perspectives, this review provides a comprehensive framework for understanding how redox imbalance reshapes developmental signaling and for identifying redox-targeted approaches to prevent and treat FASD.

## 2. Ethanol-Induced Redox Dysregulation in Developing Embryos

**Figure 1:**
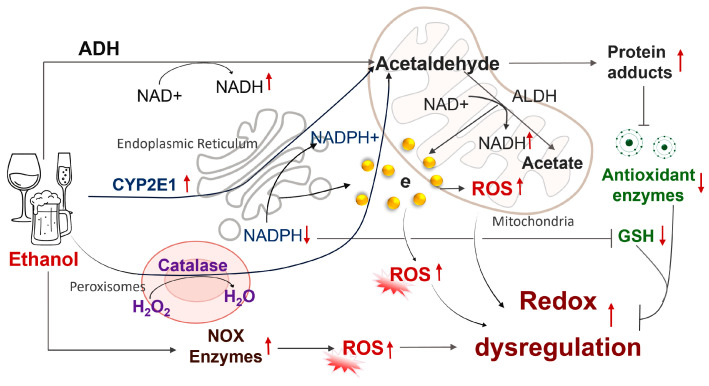
Prenatal ethanol exposure increases ROS production and suppresses antioxidant defenses, leading to redox dysregulation in the developing embryos. Prenatal ethanol exposure increases ROS generation through ethanol metabolism by alcohol dehydrogenase (ADH), CYP2E1, and catalase. NADPH oxidases (NOX) also contribute to ROS production. Prenatal ethanol exposure also reduces endogenous antioxidant capacity, including glutathione (GSH), SOD, CAT, and GPx. Together, excessive ROS and impaired antioxidant defenses disrupt redox homeostasis, leading to redox dysregulation in embryonic tissues. Upward arrows represent upregulation and downward arrows represent inhibition.

When pregnant women consume alcohol, it readily crosses the placenta, allowing fetal blood alcohol concentrations to equilibrate rapidly with maternal levels [22]. Ethanol generates excessive ROS through multiple biochemical pathways (Figure 1), including the alcohol dehydrogenase (ADH) pathway, the microsomal ethanol oxidizing system (MEOS), the catalase pathway, and NADPH oxidase (NOX) pathways [13,23,24,25,26,27,28,29,30]. In addition, ethanol depletes endogenous antioxidants, further compromising the embryo’s ability to maintain redox homeostasis. The resulting imbalance between ROS production and the embryo’s antioxidant defenses plays a central role in the pathogenesis of FASD [31,32]. This section first describes the major pathways of ROS generation and then examines how ethanol-induced ROS accumulation and antioxidant depletion disrupt redox homeostasis in developing embryos.

### 2.1. Pathways of Ethanol Metabolism and ROS Production

#### 2.1.1. Alcohol Dehydrogenase (ADH) Pathway

Ethanol is initially oxidized to acetaldehyde by ADH and subsequently converted to acetate by aldehyde dehydrogenase (ALDH2). Both reactions generate reduced nicotinamide adenine dinucleotide (NADH), thereby elevating the NADH/NAD^+^ ratio. This redox shift alters the mitochondrial respiratory chain, resulting in enhanced electron leakage during electron transfer and subsequent ROS production [23,33]. Excess NADH also disrupts redox homeostasis by inhibiting fatty acid β-oxidation. These metabolic disturbances impair mitochondrial membrane potential, promote ROS accumulation, and ultimately trigger oxidative stress and metabolic dysregulation [23,33].

#### 2.1.2. Microsomal Ethanol-Oxidizing System (MEOS)

The MEOS is an alternative pathway for ethanol metabolism that primarily occurs in the endoplasmic reticulum. Cytochrome P450 2E1 (CYP2E1), the key enzyme in MEOS, catalyzes the oxidation of ethanol to acetaldehyde. This reaction utilizes electrons from NADPH, which are transferred to CYP2E1. A feature of CYP2E1 is its high degree of catalytic uncoupling, in which electrons are only partially coupled to ethanol oxidation, allowing them to react prematurely with molecular oxygen and generate the superoxide anion (O^2−^). These superoxide anions are subsequently converted to hydrogen peroxide (H_2_O_2_) and other ROS, making the MEOS a major contributor to ethanol-induced oxidative stress. Chronic alcohol consumption markedly induces CYP2E1 expression, enhances its catalytic activity, resulting in persistent oxidative stress [26,34]. Moreover, CYP2E1 metabolizes polyunsaturated fatty acids and various xenobiotics, generating lipid radicals and hydroperoxides that initiate lipid peroxidation and further amplify oxidative damage and dysregulation [25,26,35].

#### 2.1.3. Catalase Pathway

The catalase pathway is primarily localized in peroxisomes and oxidizes ethanol using H_2_O_2_ as an electron acceptor. Under normal physiological conditions, the pathway accounts for only a small fraction of total ethanol metabolism. However, its contribution increases during sustained alcohol exposure, when intracellular H_2_O_2_ levels are elevated. During this process, catalase converts ethanol to acetaldehyde while consuming H_2_O_2_ [29,36]. Acetaldehyde is a highly reactive metabolite that disrupts mitochondrial function, enhances electron leakage from the respiratory chain, and stimulates secondary ROS production. In addition, acetaldehyde forms adducts with proteins and antioxidant enzymes, impairing their activity and weakening cellular antioxidant defenses. Together, these effects amplify ROS accumulation beyond the peroxisome and contribute to redox dysregulation in developing tissues. Thus, although catalase-mediated ethanol oxidation is a relatively minor metabolic route, the acetaldehyde it produces can indirectly drive oxidative damage and reinforce ethanol-induced redox imbalance during fetal development.

#### 2.1.4. NADPH Oxidase (NOX) Pathways and Other ROS-Generating Systems

In addition to classical ethanol-metabolizing enzymes, excessive ROS production can be induced by a non-metabolic ROS-generating system, particularly NADPH oxidases (NOX). Several NOX isoforms, including NOX2 and NOX4, are expressed in embryonic and placental tissues [37,38]. Prenatal ethanol exposure has been shown to enhance the activity and expression of these NOX enzymes, leading to increased ROS generation and contributing substantially to ethanol-induced oxidative stress [13]. Ethanol exposure also perturbs cellular iron homeostasis, increasing intracellular free iron levels, which catalyze the formation of highly reactive hydroxyl radicals via Fenton reactions [21]. In parallel, reactive intermediates of ethanol metabolism form covalent adducts with proteins, lipids, and DNA, impairing mitochondrial enzymes and antioxidant systems, thereby amplifying ROS formation [30,39].

### 2.2. Ethanol-Induced Redox Imbalance in the Developing Embryos

#### 2.2.1. Ethanol Exposure Results in Excessive Generation of ROS in Developmental Embryos

Although the maternal liver metabolizes the majority of ingested ethanol, a portion readily crosses the placenta and diffuses directly into embryonic tissues. Limited fetal metabolic capacity at the early stage of embryonic development prolongs ethanol exposure, promoting sustained ROS generation that can overwhelm endogenous antioxidant defenses and induce oxidative stress [13,15]. For example, Devi et al. reported elevated malondialdehyde (MDA) levels following prenatal ethanol exposure, indicating heightened oxidative stress [40]. Consistently, Chen and Sulik further demonstrated that ethanol exposure promoted iron-mediated free radical formation and superoxide generation in neural crest cells (NCCs), leading to oxidative damage and cell death [17,21]. In addition, Dong et al. and Hill et al. showed that ethanol increased NOX-dependent ROS production, which contributed to apoptosis in mouse embryos [38,41]. Together, these findings support a central role for ROS in mediating ethanol-induced developmental defects characteristic of FASD.

#### 2.2.2. Ethanol Exposure Suppresses and Depletes Endogenous Antioxidants in Developing Embryos

Ethanol exposure can also suppress and deplete endogenous antioxidants in developing embryos, either directly or through its metabolite, acetaldehyde. Prenatal alcohol exposure has been shown to impair the antioxidant defense system by reducing the expression and activity of key antioxidant enzymes. Drever et al. reported that prenatal ethanol exposure significantly decreased the mRNA expression of superoxide dismutase (SOD), glutathione peroxidase (GPx), and catalase (CAT) in the fetal mouse brain [42]. Acetaldehyde, a highly electrophilic metabolite of ethanol, forms covalent adducts with nucleophilic amino acid residues, particularly lysine ε-amino groups, through Schiff base formation [43,44]. These adducts can become stabilized via secondary reactions or by co-modification with lipid peroxidation products such as malondialdehyde, resulting in stable and functionally disruptive protein modifications. Acetaldehyde-mediated adduction alters enzyme conformation and activity, impairs mitochondrial and antioxidant systems, and contributes to sustained redox imbalance. Consistent with these findings, Sogut et al. demonstrated that gestational ethanol exposure significantly reduced GPx activity and altered SOD and CAT activity in the fetal rat brain, indicating disruption of enzymatic antioxidant defenses [45]. Yan et al. further showed that ethanol exposure suppressed antioxidant enzyme activity in NCCs [46]. Collectively, these findings demonstrate that ethanol exposure disrupts endogenous antioxidant defenses in developing embryos, thereby increasing susceptibility to oxidative damage (Figure 1).

## 3. Molecular Networks of Redox Regulation Disrupted by Prenatal Ethanol Exposure in FASD Pathogenesis

**Figure 2:**
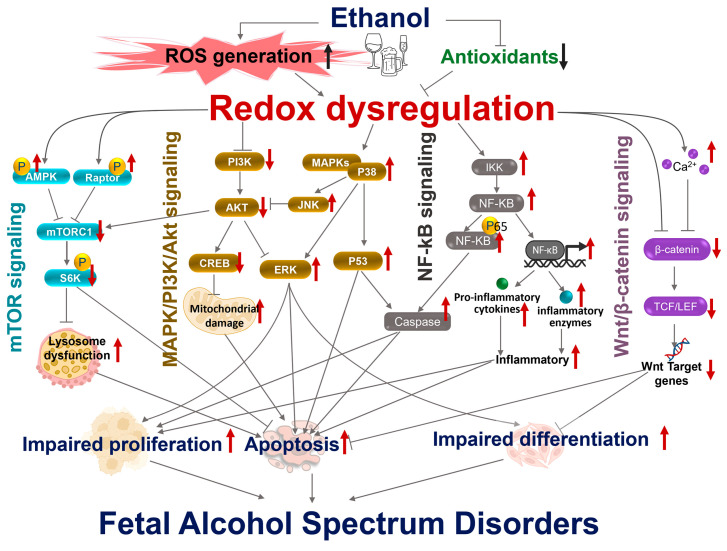
Prenatal ethanol exposure induces redox dysregulation that disrupts multiple redox-sensitive signaling pathways, including MAPK, PI3K/Akt, NF-κB, Wnt/β-catenin, and mTOR, leading to widespread developmental abnormalities characteristic of FASD. Red upward arrows indicate ethanol-induced upregulation; red downward arrows indicate ethanol-induced downregulation. AMPK: AMP-activated protein kinase; Raptor: Regulatory associated protein of mTOR; mTORC1: Mechanistic target of rapamycin complex 1; S6K: Ribosomal protein S6 kinase; PI3K: Phosphoinositide 3-kinase; AKT: Protein kinase B; CREB: cAMP response element-binding protein; MAPKs: Mitogen-activated protein kinases; P38: p38 mitogen-activated protein kinase; JNK: c-Jun N-terminal kinase; ERK: Extracellular signal-regulated kinase; P53: Tumor protein p53; IKK: IκB kinase; NF-κB: Nuclear factor kappa-light-chain-enhancer of activated B cells; Caspase: Cysteine-aspartic proteases; β-catenin: Beta-catenin; TCF/LEF: T-cell factor/Lymphoid enhancer-binding factor.

Redox homeostasis is tightly regulated during embryonic development, as physiological levels of ROS function as signaling molecules that coordinate proliferation, differentiation, migration, and survival. Prenatal alcohol exposure disrupts this balance by promoting excessive ROS generation and weakening endogenous antioxidant defenses, resulting in sustained redox dysregulation [1,15]. Ethanol-induced redox dysregulation modifies redox-sensitive proteins and transcription factors, thereby perturbing multiple interconnected signaling pathways that govern developmental processes (Table 1). Among these pathways, the Nrf2/Keap1 axis serves as a master regulator of antioxidant gene expression [47,48,49]. Concurrently, stress-activated kinases, including MAPKs (JNK and p38), are aberrantly activated by oxidative signals, promoting apoptosis and disrupting normal differentiation programs [50]. The PI3K/Akt pathway, which supports cell survival and growth, is also sensitive to redox changes [50]. In parallel, activation of NF-κB signaling enhances inflammatory responses and further amplifies oxidative injury [51]. Redox perturbations additionally interfere with Wnt/β-catenin signaling, a pathway essential for neural patterning and tissue morphogenesis, thereby impairing developmental signaling cues [52,53,54]. mTOR signaling also serves as a central nexus that balances growth and survival signaling in response to oxidative stress [55,56,57]. These redox-sensitive pathways do not operate in isolation; rather, they form an integrated signaling network in which oxidative imbalance propagates through reciprocal crosstalk mechanisms. Disruption of this network alters gene expression, mitochondrial, endoplasmic reticulum, and lysosomal function, and cellular processes, ultimately contributing to the structural, functional, and neurobehavioral abnormalities characteristic of FASD (Figure 2).

### 3.1. Nrf2/Keap1 Signaling and Antioxidant Gene Regulation

Nuclear factor erythroid 2-related factor 2 (Nrf2)/Keap1 pathway is a central redox-sensitive regulatory system that coordinates cellular defense against ethanol-induced oxidative stress [1,16,46,47,48]. Under normal conditions, Nrf2 is retained in the cytoplasm by Keap1 and degraded via the Keap1–Cul3–E3 ligase complex [58,59]. During oxidative stress, including ethanol-derived ROS, Keap1 cysteine residues are modified, releasing Nrf2. Stabilized Nrf2 accumulates and translocates to the nucleus, where it binds to antioxidant response elements (AREs) and induces the expression of detoxifying and antioxidant enzymes [60,61]. Dong and colleagues were the first to investigate Nrf2 signaling in the pathogenesis of FASD. They found that ethanol exposure in pregnant mice increases embryonic Nrf2 protein levels and ARE-binding activity, accompanied by modest increases in the transcription and protein expression of downstream antioxidant genes, reflecting an adaptive response to elevated oxidative stress that is insufficient to prevent ethanol-induced apoptosis in mouse embryos [47]. However, pharmacological activation of Nrf2 using 3H-1,2-thione (D3T) further augmented Nrf2 accumulation and ARE-binding activity, markedly upregulated Nrf2-regulated antioxidant genes, and significantly reduced ethanol-induced ROS production and apoptosis in embryos [47]. Subsequent work from the same group demonstrated that enforced expression of Nrf2 enhanced ARE-driven transcription and increased both the expression and enzymatic activities of antioxidant targets. Elevated Nrf2 levels reduced ROS generation and attenuated apoptosis in ethanol-treated NCCs [48]. In addition, pharmacological inducers of Nrf2, including tert-butylhydroquinone (tBHQ) and sulforaphane (SFN), stimulated Nrf2 signaling and downstream antioxidant expression, thereby protecting NCCs from ethanol-induced oxidative stress and apoptotic cell death [46,49]. Collectively, these studies support a protective role for Nrf2-mediated antioxidant defense in modulating the vulnerability of developing embryos to ethanol-induced oxidative injury.

### 3.2. ROS-Modulated MAPK and PI3K/Akt Signaling Pathways

#### 3.2.1. Effects of ROS-Driven MAPK Activation on Cell Differentiation and Survival

Excessive ROS generation following prenatal ethanol exposure aberrantly activates multiple mitogen-activated protein kinase (MAPK) pathways, including ERK, JNK, and p38. Under physiological conditions, ERK activation is transient and supports neuronal survival, proliferation, and differentiation [62]. In contrast, sustained p38/JNK activation is linked to stress responses and apoptotic outcomes. Sustained oxidative stress induced by ethanol exposure preferentially activates the stress-responsive MAPKs, particularly JNK and p38 [50,63,64]. Ku et al. demonstrated that ethanol exposure increased intracellular ROS levels and activated ERK, JNK, and p38 MAPKs in HT22 mouse hippocampal neurons [64]. Notably, ERK activation was transient, whereas p38 activation became sustained with prolonged exposure. Pharmacological inhibition of p38 using SB203580 significantly protected against ethanol-induced cell death, whereas inhibition of ERK or JNK did not confer comparable protection. These findings indicate that sustained p38 activation plays a dominant role in mediating ethanol-induced oxidative injury in hippocampal neurons [64]. Moon et al. reported that ethanol exposure increased phosphorylation of both c-JNK and p38, accompanied by enhanced p53 phosphorylation, elevated expression of apoptotic markers, and cell-cycle arrest. Pharmacological inhibition of JNK or p38 attenuated apoptotic signaling, supporting the involvement of stress-activated MAPKs and p53-mediated apoptosis in this model [65]. Consistently, Yuan et al. reported that ethanol exposure up-regulated the E3 ubiquitin ligase Siah1, which promoted phosphorylation of p38 MAPK and stabilization and activation of p53 in NCCs. Knockdown of Siah1 or inhibition of p38 significantly reduced p53 activation and ethanol-induced apoptosis, indicating a mechanistic cascade involving Siah1-p38-p53 signaling [50]. Collectively, these studies demonstrate that excessive ROS induced by ethanol leads to sustained JNK/p38 activation and pro-apoptotic transcription factor phosphorylation, thereby impairing neural crest cells or neuronal survival during development [50,63,64].

#### 3.2.2. Activation of PI3K/Akt Signaling by ROS in Cell Proliferation and Apoptosis

The phosphoinositide 3-kinase (PI3K)/Akt pathway is a central regulator of cellular survival, growth, and migration [66]. Activation of Akt through phosphorylation at Thr308 and Ser473 promotes downstream signaling that inhibits pro-apoptotic proteins and supports transcriptional programs necessary for cell survival [67]. Chong et al. emphasized that Akt functions as a critical survival kinase in the nervous system, counteracting inflammatory and apoptotic injury by stabilizing mitochondrial function and suppressing pro-death signaling cascades. Akt also modulates transcription factors such as CREB and inhibits components of stress-activated pathways, thereby maintaining cellular homeostasis [68]. Disruption of this survival axis increases vulnerability to oxidative injury. In the context of ethanol exposure, suppression of PI3K/Akt signaling has been implicated in cellular damage [69]. Kumar et al., in their review of ethanol neurotoxicity in the developing cerebellum, describe attenuation of pro-survival pathways, including PI3K/Akt, as part of the mechanistic framework underlying ethanol-induced neuronal loss [70]. Experimental support for ethanol-mediated impairment of Akt signaling has also been demonstrated in animal models. Ren et al. showed that chronic ethanol exposure in the cerebral cortex significantly decreased phosphorylated Akt levels [71]. In addition, Liu et al. discovered that chronic ethanol exposure induced an inactivation of PI3K/Akt in both PC12 cells and primary cortical neurons [72]. This reduction was accompanied by increased oxidative stress markers, mitochondrial dysfunction, and enhanced apoptotic susceptibility, consistent with compromised survival signaling [71,73]. Importantly, PI3K/Akt signaling does not operate in isolation but functionally intersects with MAPK pathways to regulate cell fate decisions. Under sustained oxidative stress, increased activation of stress-responsive kinases such as JNK and p38, together with reduced Akt phosphorylation, shifts the intracellular balance away from pro-survival signaling toward pro-apoptotic pathways. Consistent with this, ethanol-induced oxidative stress disrupts both PI3K/Akt activity and its crosstalk with MAPKs, thereby promoting a signaling environment that contributes to neuronal vulnerability and developmental injury.

### 3.3. NF-κB Signaling as a Redox-Sensitive Transcriptional Integrator in Ethanol-Induced Developmental Injury

The nuclear factor-κB (NF-κB) pathway is a central redox-sensitive transcriptional regulator that integrates oxidative stress, inflammatory signaling, cell survival, and neurodegenerative processes [74,75]. Under basal conditions, NF-κB dimers, most commonly p65 (RelA)/p50, are retained in the cytoplasm through association with inhibitor of κB (IκB) proteins. Activation of the IκB kinase (IKK) complex triggers phosphorylation and ubiquitin-mediated degradation of IκBα, permitting NF-κB nuclear translocation and transcriptional activation of target genes [75]. As reviewed by Singh and Singh [74], NF-κB signaling exerts context-dependent effects in the nervous system, contributing to both neuronal survival and degeneration, underscoring that its function extends well beyond classical inflammatory responses. ROS act as signaling mediators capable of modulating NF-κB activation [74,75,76]. Akhtar et al. demonstrated that acute maternal oxidant exposure increases fetal brain susceptibility to oxidative stress and inflammatory activation, linking redox imbalance to NF-κB–associated responses in developmental contexts [76]. Importantly, NF-κB regulates not only cytokines and inflammatory enzymes but also genes involved in oxidative stress adaptation, apoptotic regulation, synaptic plasticity, and neurodevelopmental processes [74]. Thus, NF-κB functions as a redox-responsive transcriptional integrator coordinating multiple stress-adaptive and injury-related gene programs.

Evidence from experimental studies of ethanol exposure supports sustained neuroimmune and redox-sensitive NF-κB activation. Kane et al. showed that postnatal ethanol exposure in a mouse model of FASD alters cerebellar inflammatory gene expression and produces persistent neuroinflammatory changes extending beyond the exposure period [77]. Qin and Crews further demonstrated that chronic ethanol exposure triggers NOX-ROS-dependent activation of NF-κB signaling pathways, which play important roles in ethanol-induced neuroinflammation and neurodegeneration, implicating innate immune signaling upstream of NF-κB in ethanol-mediated brain injury [78]. In adolescent intermittent ethanol models, Vetreno et al. reported persistent hippocampal neuroimmune activation in adulthood, accompanied by increased NF-κB p65 activation and long-term reductions in hippocampal neurogenesis; these effects were attenuated by exercise or indomethacin treatment [79]. Together, these findings link ethanol-induced NF-κB–associated signaling to persistent neuroinflammation, impairment of neural progenitor populations, and long-lasting alterations in brain plasticity. Antioxidant intervention studies further support a functional role for ROS-dependent NF-κB activation in ethanol teratogenicity. Peng et al. demonstrated that ascorbic acid suppresses ethanol-induced ROS production, inhibits NF-κB activation, and prevents growth retardation and microencephaly in *Xenopus laevis* embryos [51]. These findings strengthen the causal relationship between oxidative stress, NF-κB activation, and developmental abnormalities. Collectively, these studies demonstrate that NF-κB signaling promotes persistent neuroinflammation in FASD by converting transient ethanol-induced oxidative stress into sustained inflammatory gene expression, supporting a model in which ROS-dependent NF-κB activation serves as a key driver of persistent neuroinflammation and long-term neuropathology in FASD.

### 3.4. Disruption of Wnt/β-Catenin Signaling in Ethanol-Induced Developmental Impairment

Multiple studies demonstrate mechanistic cross-talk between redox regulation and Wnt/β-catenin signaling [80]. ROS-dependent modulation of Wnt/β-catenin signaling has been shown to influence cellular behavior through combined in vitro and in silico approaches [80], supporting the concept that redox status is an upstream regulator of canonical Wnt signaling. Wnt/β-catenin signaling plays essential roles throughout nervous system development. Canonical Wnt signaling regulates neuronal maturation, dendritic development, synapse formation, and synaptic plasticity [81]. More broadly, Wnt pathways govern neural patterning, neuronal connectivity, and circuit assembly in the vertebrate nervous system [82,83]. In the canonical pathway, Wnt ligands bind Frizzled receptors and LRP5/6 co-receptors, leading to inhibition of the β-catenin destruction complex, stabilization of cytoplasmic β-catenin, and its nuclear translocation to interact with TCF/LEF transcription factors to regulate gene expression [84]. Beyond development, activation of Wnt/β-catenin signaling in the adult brain has been shown to modulate neurodegeneration and cognitive impairment in an Alzheimer’s disease mouse model [85], underscoring its functional importance in neural maintenance.

Ethanol exposure has been shown to disrupt the Wnt/β-catenin signaling pathway. In an avian model of fetal alcohol spectrum disorders, ethanol exposure depletes nuclear β-catenin, reducing β-catenin-mediated transcription and downregulating critical downstream genes essential for neural crest development and survival, including Slug, FoxD3, and Wnt6 [52]. Notably, forced β-catenin expression, as well as suppression of the ethanol-induced Ca^2+^ transient by using Bapta-AM, effectively prevented neural crest cell loss in ethanol-exposed embryos [52]. Ethanol-mediated disruption of Wnt/β-catenin signaling has also been demonstrated in human brain endothelial cells (hCMEC/D3) [86]. Activation of Wnt signaling through known Wnt activators (i.e., LiCl or Wnt3a) attenuates ethanol-induced cellular dysfunction, including increased endothelial permeability in brain microvascular cells [86], which is consistent with the established role of Wnt/β-catenin signaling in regulating blood–brain barrier formation and endothelial differentiation during neural development, as extensively described by Ciani and Salinas [82]. In addition, findings in zebrafish and chicks demonstrate an evolutionarily conserved calcium-dependent mechanism of ethanol-induced neurotoxicity, including CaMKII activation, destabilization of transcriptionally active β-catenin, and increased apoptosis, all of which are associated with craniofacial defects [52,53,54,87,88,89]. These studies support a model in which ethanol-induced calcium dysregulation suppresses β-catenin signaling and compromises neural crest cell survival [52,53].

### 3.5. mTOR Signaling in Ethanol-Induced Neural Crest Cell Impairment

The mechanistic target of rapamycin (mTOR) is a central serine/threonine kinase that integrates growth factor, nutrient, and cellular stress signals to regulate metabolism, protein synthesis, cell survival, and cytoskeletal dynamics. mTOR functions through two distinct complexes: mTORC1, which controls translational output and ribosomal biogenesis, and mTORC2, which modulates cytoskeletal organization and cell survival. Both complexes are sensitive to upstream nutrient and stress regulators such as the PI3K/AKT axis, oxidative stress, and energy-sensing kinases, AMP-activated protein kinase (AMPK) [90]. Oxidative stress profoundly influences cellular metabolism and mTOR activity. ROS can activate stress-responsive kinases that converge on PI3K/AKT and AMPK pathways, ultimately modulating mTORC1 activity and downstream translational programs [90]. In this framework, mTORC1 functions as a metabolic rheostat that balances growth and survival signaling in response to environmental stressors.

Ethanol exposure perturbs these regulatory networks in developing tissues. Chronic prenatal alcohol exposure alters mTOR signaling in the fetal hippocampus, characterized by reduced phosphorylation of mTOR and dysregulation of its downstream targets [55]. In neural crest contexts, ethanol-induced suppression of mTOR signaling contributes to apoptotic programs. Ethanol exposure has been shown to increase the phosphorylation of AMPK and Raptor, an essential component of mTORC1, and phosphorylation of Raptor is indicative of mTORC1 suppression. Functional gain- or loss-of-function approaches targeting AMPK, S6K, or TORC1 demonstrated that attenuation of this pathway prevented ethanol-induced suppression of rRNA synthesis and blocked p53-dependent apoptosis. These findings establish the mTORC1/S6K signaling as a critical survival axis in cranial neural crest cells and identify AMPK-mediated mTORC1 suppression as one of the central mechanisms underlying alcohol-induced apoptosis in neural crest cells [56]. Consistent with these observations, genetic studies in zebrafish revealed that ethanol exposure significantly increased neural crest cell death and craniofacial defects in PDGF receptor alpha (pdgfra) mutants. Importantly, mTOR signaling enhancement partially rescued ethanol-induced craniofacial defects in this model [57]. Taken together, these studies position mTOR signaling at the nexus of oxidative stress, growth factor signaling, and ethanol toxicity.

In summary, ethanol-induced oxidative stress activates a network of redox-sensitive signaling pathways that collectively disrupt critical developmental processes (Table 1 and Figure 2). The Nrf2/Keap1 axis serves as the primary antioxidant defense, inducing cytoprotective and detoxifying genes in response to elevated ROS. When oxidative stress persists, MAPK family members (ERK, JNK, p38) and the PI3K/Akt pathway transduce redox signals to regulate the balance between adaptive survival responses and apoptosis. Sustained ROS also stimulates NF-κB signaling, enhancing pro-inflammatory gene expression and reinforcing a feed-forward inflammatory state. Oxidative stress also perturbs Wnt/β-catenin signaling, disrupting transcriptional programs required for proper developmental progression. ROS-mediated activation of AMPK also suppresses mTORC1 activity, leading to reduced S6K-dependent ribosomal biogenesis and promoting p53-mediated apoptosis. Together, these pathways constitute an integrated redox-responsive network in which ROS, antioxidant defenses, and developmental regulation are tightly interconnected, and their coordinated disruption under ethanol exposure provides a mechanistic framework for developmental pathology in FASD.

## 4. Multi-Level Oxidative Damage and Developmental Consequences of Ethanol-Induced Redox Dysregulation

**Figure 3:**
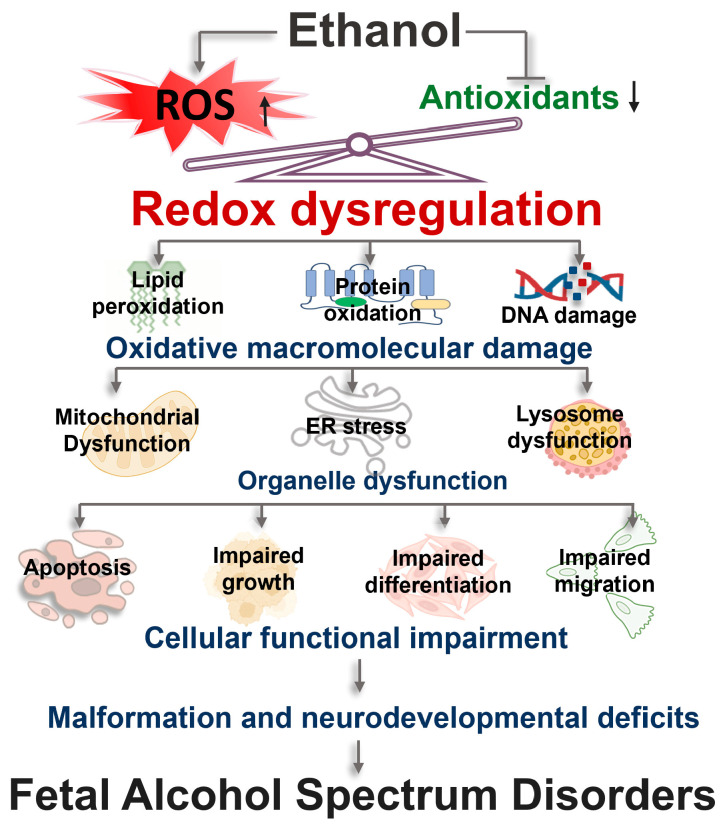
Oxidative stress induced by prenatal ethanol exposure causes oxidative damage to cellular macromolecules and impairs cell survival, proliferation, and differentiation and migration, resulting in structural abnormalities and neurodevelopmental deficits associated with FASD.

Ethanol-induced redox dysregulation acts as a primary biochemical trigger, initiating a sequence of multi-level oxidative damage that disrupts fetal development (Figure 3). At the molecular level, this oxidative imbalance drives extensive DNA damage, protein oxidation, and lipid peroxidation. These chemical insults and disrupted redox-sensitive signaling networks damage organelles, manifesting as mitochondrial dysfunction, ER stress, and lysosomal impairment. At the cellular level, the resulting redox imbalance disrupts critical developmental processes, including proliferation, differentiation, and migration, while triggering aberrant apoptotic pathways. Ultimately, this multi-level oxidative cascade underlies the structural malformations and neurodevelopmental deficits characteristic of FASD.

### 4.1. Oxidative Macromolecular Damage

ROS generated during alcohol metabolism induce widespread oxidative damage to major cellular macromolecules. Elevated ROS levels induce lipid peroxidation, leading to membrane damage and altered lipid signaling. ROS also promote protein carbonylation, nitration, and aggregation, thereby compromising protein structure and function. In addition, ROS damages nucleic acids by causing strand breaks and oxidative base modifications [91,92]. In neurons, excessive ROS induces lipid peroxidation and protein oxidation, impairing membrane integrity, mitochondrial function, and protein turnover [93]. Activated microglia and astrocytes generate nitric oxide (NO) and superoxide, which combine to form peroxynitrite, a highly reactive species that induces DNA fragmentation and lipid oxidation [91]. The accumulation of oxidative damage compromises glial antioxidant defenses and disrupts essential cellular functions, including calcium homeostasis, glutamate uptake, and trophic factor secretion. Endothelial cells are similarly vulnerable, where ROS-mediated oxidation and nitration of amino acid residues in proteins, such as the formation of 3-nitrotyrosine from tyrosine nitration, and protein carbonylation contribute to protein cross-linking, structural alterations, functional impairment, and increased proteolytic susceptibility. At the DNA level, highly reactive hydroxyl radicals (·OH) and peroxynitrite induce base oxidation, DNA strand breaks, and formation of lesions such as 8-hydroxy-2′-deoxyguanosine (8-OHdG) [94].

ROS-induced macromolecular damage contributes to the disruption of key molecular signaling networks. Early oxidative events include the nuclear accumulation of Ref-1/APE1, which regulates the DNA-binding of transcription factors such as NF-κB and AP-1 [94,95]. ROS also activate the Nrf2-dependent antioxidant response, enhancing the expression of cytoprotective genes. It can also disrupt the nucleoredoxin (NRX)–Disheveled (DVL) interaction, thereby facilitating Wnt/β-catenin signaling [80,96,97]. As lipid peroxidation progresses and reactive aldehydes such as 4-hydroxynonenal (4-HNE) and MDA accumulate, they form exocyclic DNA adducts with mutagenic potential and modulate MAPK signaling, including JNK, ERK, and p38 [98]. ROS and lipid peroxidation also stimulate inflammatory signaling, including activation of the NF-κB signaling pathway, and induction of pro-inflammatory mediators [99]. Oxidative modification of key regulatory proteins such as PTEN further alters PI3K/Akt signaling [100]. Collectively, ethanol-induced ROS results in macromolecular damage to lipids, proteins, and DNA, and subsequent disruption of redox-sensitive signaling networks, ultimately contributing to cellular dysfunction and the pathogenesis of FASD.

### 4.2. Redox Dysregulation Results in Organelle Dysfunction

#### 4.2.1. Mitochondrial Dysfunction Induced by ROS

Excessive ROS disrupts mitochondrial structure and function, compromising cellular energy production, calcium homeostasis, and survival [101]. Mitochondria are both a major source and target of ROS. Elevated ROS levels damage mitochondrial membranes and components of the electron transport chain, impairing electron flow, reducing ATP synthesis, and promoting electron leakage that further amplifies ROS generation. This creates a self-reinforcing cycle of oxidative stress and mitochondrial dysfunction. Mitochondria also play a central role in intracellular calcium regulation. However, ROS disrupts mitochondrial calcium handling by impairing calcium transport mechanisms, leading to mitochondrial calcium overload. Excessive intracellular calcium is highly cytotoxic and represents a key mediator of neuronal injury and death [102]. Ethanol exposure further exacerbates this process by promoting calcium dysregulation and oxidative stress, thereby enhancing cellular vulnerability [103]. The combined effects of oxidative damage and calcium overload promote the opening of the mitochondrial permeability transition (MPT) pore, resulting in the loss of mitochondrial membrane potential, impaired oxidative phosphorylation, and release of pro-apoptotic factors, such as cytochrome c, into the cytosol [103]. This triggers activation of the intrinsic apoptotic pathway through caspase activation. ROS also regulates mitochondrial integrity through redox-sensitive signaling pathways. Activation of stress-responsive kinases, including c-Jun N-terminal kinase (JNK), has been shown to promote mitochondrial dysfunction and apoptosis in a ROS-dependent manner [104]. JNK can enhance mitochondrial outer membrane permeabilization and facilitate the release of pro-apoptotic factors. In parallel, redox signaling modulates the intrinsic apoptotic machinery by influencing the balance of Bcl-2 family proteins [105]. Pro-apoptotic proteins such as Bax and Bak are activated and translocated to the mitochondrial membrane, while anti-apoptotic proteins such as Bcl-2 are suppressed, collectively promoting mitochondrial membrane destabilization. Consistent with this mechanism, ethanol exposure has been shown to induce Bax translocation, mitochondrial membrane depolarization, and increased ROS production, effects that can be attenuated by antioxidant treatment or neurotrophic support [106]. Together, these findings demonstrate that ROS-induced mitochondrial dysfunction serves as a central mechanism linking oxidative stress to calcium dysregulation and activation of intrinsic apoptotic pathways, thereby contributing to cellular injury in FASD.

#### 4.2.2. ER Stress Induced by ROS

The endoplasmic reticulum (ER) is essential for maintaining cellular proteostasis, as it governs protein folding, post-translational modification, and trafficking. Disruption of ER homeostasis leads to the accumulation of unfolded or misfolded proteins, triggering the ER stress, which shifts signaling toward apoptosis and cellular injury [107]. Ethanol-induced oxidative stress is a key contributor to ER dysfunction. Elevated ROS generated during ethanol exposure promotes oxidative post-translational modifications of proteins within the ER, resulting in protein misfolding and impaired folding efficiency. In parallel, oxidative stress disrupts the function of critical ER chaperones and folding enzymes, including glucose-regulated protein 78 (GRP78), protein disulfide isomerase (PDI), and other redox-sensitive components of the protein folding machinery, thereby compromising ER proteostasis and amplifying protein misfolding [108,109]. These events lead to activation of canonical UPR signaling pathways, including PERK, eIF2α, IRE1α, and ATF6. Experimental studies have shown that ethanol exposure induces ER stress markers, including GRP78, CHOP, phosphorylated PERK, eIF2α, and IRE1α, along with increased LC3-II levels, indicating coordinated activation of ER stress and autophagy pathways in ethanol-mediated neurotoxicity [108,109]. ER function is also critically dependent on tightly regulated Ca^2+^ homeostasis, which is required for proper protein folding and chaperone activity. Ethanol perturbs intracellular Ca^2+^ signaling, further exacerbating ER stress. In neural crest cells, ethanol has been shown to induce a rapid Ca^2+^ transient, leading to intracellular Ca^2+^ release [88,89], which may impair ER folding capacity and promote ER stress–mediated apoptosis.

#### 4.2.3. Lysosome Dysfunction Induced by ROS

The autophagy–lysosome pathway plays a critical role in maintaining cellular homeostasis by degrading oxidized proteins and damaged organelles, including dysfunctional mitochondria [108,110]. Under physiological conditions, autophagy serves as an adaptive response to oxidative stress, facilitating the clearance of ROS–damaged cellular components. However, excessive ROS production induced by ethanol exposure disrupts autophagy–lysosome function. Ethanol-induced oxidative stress frequently impairs autophagic flux, leading to the accumulation of autophagosomes and defective cargo clearance [108]. This impairment is often associated with lysosomal dysfunction, including reduced degradative capacity and altered lysosomal activity, which compromises the final steps of autophagy. As a result, damaged proteins and organelles accumulate, contributing to cellular stress and injury. Emerging evidence indicates that disruption of the autophagy-lysosome pathway is a key mechanism in alcohol-induced neurotoxicity. For example, deficiency of the E3 ubiquitin ligase Parkin exacerbates alcohol-induced tissue injury, which is associated with impaired autophagy and mitochondrial quality control [111]. Conversely, pharmacological activation of NRF2 has been shown to restore mitophagy initiation, suppress inflammasome activation, and improve cognitive function following chronic alcohol exposure [112]. Similarly, modulation of autophagy has been implicated in the neuroprotective effects of compounds such as puerarin, which alleviates alcohol-induced cognitive impairment by improving autophagic activity [113]. Oxidative stress is closely linked to lysosomal dysfunction through redox imbalance. Ethanol exposure has been shown to deplete glutathione (GSH) and disrupt cellular redox homeostasis, thereby enhancing oxidative stress and contributing to lysosomal impairment [114]. In addition, transcriptional regulation of lysosomal biogenesis is affected by alcohol exposure. The transcription factor EB (TFEB), a master regulator of lysosomal and autophagy gene expression, has been reported to be reduced in association with alcohol exposure in some experimental contexts, accompanied by increased protein aggregation and apoptotic signaling in the brain [115]. Furthermore, activation of Toll-like receptor 4 (TLR4) signaling has been shown to impair both the ubiquitin–proteasome system and the autophagy–lysosome pathway in response to ethanol exposure, leading to accumulation of damaged proteins and altered lysosomal function [116]. Collectively, these findings indicate that ethanol-induced oxidative stress disrupts the autophagy–lysosome pathway at multiple levels. This dysregulation leads to the accumulation of damaged cellular components, amplification of oxidative stress, and activation of apoptotic pathways, thereby contributing to alcohol-mediated brain injury and neurodevelopmental deficits [108,117].

### 4.3. Redox Dysregulation Impairs Cellular Integrity and Functions in Developing Tissues

#### 4.3.1. Redox Dysregulation Contributes to Apoptosis in Developing Tissues

Excessive ROS can induce apoptotic cell death in developing tissues through multiple interconnected molecular mechanisms [14,118,119]. Oxidative stress disrupts mitochondrial integrity, leading to loss of mitochondrial membrane potential, release of cytochrome c, and activation of caspase-dependent apoptotic pathways, which represent central features of ethanol-induced developmental toxicity [14,103]. In neuronal models, oxidative stress is closely associated with aberrant re-entry into the cell cycle, as evidenced by the upregulation of cell cycle–related proteins, such as cyclins and cyclin-dependent kinases, which precedes apoptotic cell death [120]. Neural crest cells are particularly vulnerable to ethanol-induced oxidative stress. In vitro studies demonstrate that ethanol exposure increases superoxide production and induces apoptosis, effects that are exacerbated by iron-mediated free radical formation and can be attenuated by antioxidant interventions [17,21]. These findings indicate that oxidative stress is a key mediator of ethanol-induced cytotoxicity in highly migratory and differentiating cell populations. In parallel, ROS accumulation is associated with impairment of endogenous antioxidant systems and activation of stress-responsive signaling pathways, including MAPKs such as ERK1/2 and JNK, which contribute to apoptotic signaling [121]. Consistent with these cellular observations, in vivo studies show that prenatal ethanol exposure elevates oxidative stress markers, disrupts mitochondrial function, and induces both apoptotic and necrotic cell death in vulnerable brain regions, including the developing cerebellum [70,103,119]. Increased lipid peroxidation products, such as malondialdehyde, together with impaired antioxidant defenses, including dysregulation of Nrf2-mediated responses, further sensitize cells to oxidative injury and apoptosis [70,121]. In addition, ethanol exposure has been shown to suppress the PI3K/Akt/mTOR pathway, reducing Akt phosphorylation and downstream anti-apoptotic signaling, including Bcl-2 activity, thereby promoting neuronal apoptosis [122]. Together, these findings demonstrate that redox imbalance is a central mechanism linking oxidative damage to apoptotic cell death across multiple developing tissues, contributing to the structural and functional abnormalities observed in FASD.

#### 4.3.2. Redox Dysregulation Disrupts Cell Proliferation, Migration, and Differentiation

In addition to inducing apoptosis, ethanol-induced redox imbalance disrupts fundamental cellular processes required for normal tissue development, including proliferation, migration, and lineage specification. ROS act as key modulators of multiple redox-sensitive molecular pathways, and excessive ROS generated during ethanol exposure perturbs these regulatory networks, resulting in abnormal developmental outcomes [14,64,103]. One major consequence of redox dysregulation is the disturbance of cellular growth and proliferation. Ethanol-induced oxidative stress interferes with MAPK signaling cascades, including p38 MAPK, which plays a critical role in regulating cell cycle progression and cellular responses to stress [64]. Disruption of these pathways alters normal proliferation dynamics and contributes to impaired tissue growth. In embryonic stem cell models, ethanol exposure has been shown to dysregulate pathways such as MAPK/ERK and Wnt/β-catenin, leading to altered lineage commitment and reduced capacity for proper tissue formation [14,123,124]. Consistent with this, studies in developing neural tissues demonstrate that ethanol exposure induces aberrant cell-cycle activity and reduces the diversity of progenitor cell populations, suggesting premature or inappropriate differentiation and depletion of the stem/progenitor pool [125]. These changes ultimately compromise tissue expansion and regenerative potential during development. Redox dysregulation also perturbs differentiation programs. Oxidative stress influences transcriptional networks that govern cell fate decisions, including pathways such as Wnt/β-catenin and mTOR, which are essential for coordinating proliferation with differentiation [14,103,123]. Ethanol-induced ROS can shift differentiation trajectories by altering the balance of lineage-specific transcription factors, thereby contributing to abnormal tissue patterning and organogenesis. Such effects have been observed across multiple developing systems, including neural and mesenchymal lineages, highlighting the broad impact of redox imbalance on developmental programming [14,103]. In addition, ethanol has been shown to impair NCC migration in vitro and in vivo, in part by up-regulating microRNA-34a (miR-34a), which targets key regulators of epithelial–mesenchymal transition (EMT), including Snail1, thereby disrupting migratory behavior [126]. Although the mechanism by which ethanol induces miR-34a was not directly defined in that study, oxidative stress has been demonstrated to activate miR-34a via PI3Kα-dependent signaling [127]. Given that ethanol exposure elevates intracellular ROS levels, these findings support a model in which ROS act upstream of miR-34a to modulate EMT-associated gene expression and cytoskeletal dynamics, thereby linking oxidative stress to impaired cell migration during development. Collectively, these findings indicate that ethanol-induced redox dysregulation disrupts multiple coordinated cellular processes, including proliferation, migration, and differentiation, leading to impaired developmental patterning and the structural and functional abnormalities characteristic of FASD.

### 4.4. The Contribution of Redox Dysregulation to Structural Abnormalities in FASD

Redox dysregulation is a central contributor to the structural abnormalities that characterize FASD, including craniofacial dysmorphology, microencephaly, ocular defects, limb malformations, and cardiac anomalies. Early experimental evidence demonstrated that ethanol exposure during critical periods of embryogenesis induces excessive ROS production, leading to cellular damage and congenital malformations [16]. These findings established oxidative stress as a key factor linking ethanol exposure to structural abnormalities. Craniofacial abnormalities are among the most well-recognized structural features of FASD and are highly sensitive to redox dysregulation. Neural crest cells, which contribute extensively to craniofacial structures, are especially susceptible to oxidative damage. Ethanol-induced oxidative stress impairs the development of these progenitor populations, leading to craniofacial dysmorphology. Genetic studies further support this link, demonstrating that alterations in signaling pathways that interact with cellular stress responses can modify susceptibility to ethanol-induced craniofacial defects. For example, disruption of PDGF signaling increases the severity of craniofacial abnormalities in a zebrafish model [57]. In addition, microRNA-mediated modulation of stress-responsive pathways has been shown to influence neural crest survival and craniofacial development, further highlighting the intersection between redox-sensitive processes and structural outcomes [128]. Redox dysregulation also contributes significantly to abnormalities in the development of the central nervous system. Ethanol-induced oxidative stress is associated with reduced brain growth and microencephaly, reflecting impaired expansion and structural organization of neural tissues. Experimental studies demonstrate that an elevated oxidative burden correlates with decreased brain size and developmental abnormalities, consistent with clinical manifestations observed in FASD [51]. In addition, ocular and limb malformations provide additional evidence of the broad impact of redox dysregulation on embryonic patterning. Ethanol exposure induces a range of ocular abnormalities, including microphthalmia and retinal defects, which are associated with oxidative damage during early eye development [129]. Similarly, limb malformations have been linked to oxidative stress–mediated disruption of limb bud formation and patterning [130]. Furthermore, cardiac development is also highly sensitive to oxidative perturbation. Prenatal and early postnatal ethanol exposure has been shown to result in persistent cardiac abnormalities, including myocardial injury and altered cardiac morphology. These structural defects are associated with sustained oxidative stress and activation of stress-responsive pathways, leading to long-term impairment of cardiac tissue integrity [121]. Collectively, these findings demonstrate that redox dysregulation is a critical determinant of the structural abnormalities observed in FASD. By compromising tissue integrity and disrupting the coordinated processes required for normal morphogenesis, ethanol-induced redox dysregulation induces congenital anomalies across multiple organ systems. The consistency of these effects across experimental models underscores the central role of redox dysregulation in shaping the anatomical features of FASD.

### 4.5. Redox Dysregulation and Neurodevelopmental Deficits in FASD

Prenatal ethanol exposure (PAE) generates excessive ROS in the developing brain, which disrupts neuronal and glial development and contributes to the cognitive, behavioral, and connectivity deficits characteristic of FASD [20,119]. ROS accumulation in utero alters neuronal progenitor proliferation and differentiation, impairs migration and synaptogenesis, and results in region-specific neuroanatomical abnormalities in the cortex, hippocampus, and cerebellum [64,119,125,131]. These structural perturbations underlie deficits in spatial learning, memory, motor coordination, and executive function observed in both animal models of FASD and clinical populations [7,119]. Beyond direct neuronal effects, prenatal ethanol-induced oxidative stress activates microglia and astrocytes in the fetal brain. Excessive ROS in microglia enhances the release of pro-inflammatory cytokines and induces NF-κB-dependent signaling [78,132]. ROS-mediated astrocyte activation leads to hypertrophy and dysregulation of glutathione homeostasis, which further establishes a neuroinflammatory milieu [133,134,135]. This glial-mediated neuroinflammation interferes with critical developmental processes, including neuronal migration, differentiation, and synaptic connectivity [136]. Akhtar et al. demonstrated that maternal oxidant exposure enhances susceptibility of the fetal brain to inflammatory stimuli, indicating that redox imbalance primes neuroimmune responses during development [76]. Complementing these findings, Kane and Drew [135] reported that glial activation in FASD models disrupts neural circuit formation and contributes to cognitive and behavioral deficits observed in FASD. Experimental studies also demonstrate that ROS induced by prenatal ethanol exposure and glial activation correlate with disrupted neuronal layering, impaired cortical and cerebellar organization, and decreased synaptic density, all of which persist into postnatal life [20,119]. These disruptions in early brain development contribute to long-term cognitive and behavioral impairments, consistent with observations in children with FASD [7]. In summary, prenatal ethanol-induced redox dysregulation contributes to neurodevelopmental deficits via a combination of direct neuronal oxidative damage and ROS-driven glial activation and neuroinflammation, collectively disrupting neural circuit formation, synaptic plasticity, and postnatal cognitive function [20,119,135].

## 5. Translational Implications: Targeting Redox Dysregulation for FASD Prevention and Treatment

**Figure 4:**
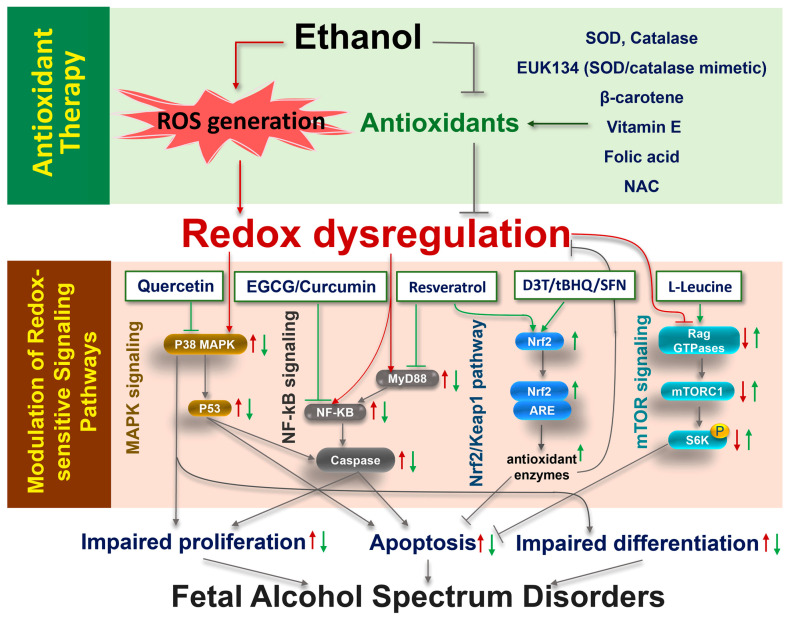
Therapeutic strategies targeting redox dysregulation in FASD. Prenatal ethanol exposure induces oxidative stress and disrupts cellular redox homeostasis (red). Ethanol-induced redox dysregulation can be restored by antioxidant-based strategies that reduce oxidative stress or by modulation of redox-sensitive signaling (green), thereby preventing or mitigating FASD-associated developmental abnormalities.

The evidence summarized in the preceding sections indicates that redox dysregulation is a central mechanism linking prenatal ethanol exposure to cellular dysfunction, structural abnormalities, and neurodevelopmental deficits in FASD. Excessive ROS generation, impaired antioxidant defenses, and disruption of redox-sensitive signaling pathways collectively contribute to the vulnerability of developing tissues to prenatal ethanol exposure. These insights provide a strong rationale for translational strategies aimed at restoring redox balance or modulating downstream signaling pathways affected by oxidative stress. Current approaches can be broadly categorized into (i) antioxidant-based interventions designed to reduce oxidative damage and reestablish cellular redox homeostasis, and (ii) targeted modulation of redox-sensitive molecular pathways that mediate ethanol-induced developmental injury (Figure 4 and Table 2).

### 5.1. Antioxidant-Based Approaches for FASD Prevention and Intervention

Given the central role of oxidative stress in ethanol-induced developmental injury and the pathogenesis of FASD, antioxidant-based interventions have been extensively investigated as a strategy to mitigate the effects of prenatal alcohol exposure (Table 2). Experimental studies have consistently demonstrated that enhancing antioxidant defenses during prenatal ethanol exposure can reduce the incidence and severity of developmental defects. Studies have shown that supplementation with exogenous SOD attenuated ethanol-induced malformations, including anterior neural tube defects, in cultured mouse embryos [16], establishing that strengthening antioxidant capacity can prevent structural abnormalities. Similarly, treatment with free radical scavengers such as SOD, catalase, and α-tocopherol (vitamin E) prevents ethanol-induced apoptosis in cranial neural crest cells, supporting the concept that vulnerable cell populations can be protected through redox stabilization [17]. Additional studies have shown that supplementation with endogenous antioxidants, vitamin E, or SOD/catalase mimetic EUK-134, reduces ethanol-induced teratogenesis in vivo [31,130]. Targeting enzymatic sources of ROS has also proven effective; inhibition of NOX activity reduces apoptosis in ethanol-exposed embryos [38].

Nutritional and pharmacologic antioxidants have also been investigated for their potential to prevent or attenuate the consequences of prenatal ethanol exposure. Maternal alcohol consumption can interfere with the placenta’s ability to transfer folate from the maternal circulation to the fetus, thereby reducing fetal folate availability [137]. Notably, folic acid supplementation has been demonstrated to reduce oxidative stress and the formation of thiobarbituric acid reactive substances (TBARS) in offspring caused by maternal ethanol consumption during pregnancy [138]. Studies have also shown that folic acid supplementation prevented ethanol-induced congenital heart defects [139]. Together, these observations suggest that maintaining adequate folate levels during pregnancy may help reduce vulnerability to ethanol-induced developmental injury and provide a potential avenue for preventive intervention. In addition to folic acid, in vitro evidence indicates that vitamin E can attenuate ethanol-induced oxidative injury in fetal rat hippocampal cultures, improving cell viability across a range of ethanol exposures, particularly at higher supplementation levels [140]. Heaton et al. reported that neonatal rats exposed to ethanol exhibited significant loss of cerebellar Purkinje cells, whereas co-administration of a high dose of vitamin E markedly reduced this neuronal loss [141]. These findings suggest a protective effect of vitamin E against ethanol-induced neurodevelopmental damage. Moreover, NAC remains one of the most extensively studied interventions; as a precursor to glutathione, it supports intracellular redox balance. It has been demonstrated that NAC supplementation reduces the incidence of ocular malformations in mouse embryos exposed to ethanol in vivo, demonstrating that restoring intracellular antioxidant capacity can prevent specific developmental defects [129].

In addition to these classical antioxidants, several bioactive compounds have shown preventive potential in experimental models. Epigallocatechin gallate (EGCG), a powerful antioxidant polyphenol primarily found in green tea, has been reported to protect against FASD, ameliorating fetal growth restriction and preventing FASD-related cognitive impairment and heart damage [142,143,144,145,146]. It also reported that cannabidiol (CBD) can prevent oxidative stress by increasing antioxidant levels and activity [147,148], reduce neuroinflammation, and ameliorate cognitive and behavioral impairments induced by prenatal ethanol exposure [149,150].

Although clinical evidence remains limited, early studies suggest that antioxidant-based interventions may confer benefits when administered during pregnancy or shortly after ethanol exposure, with reported improvements in cognitive and behavioral outcomes in some cohorts [151,152,153]. At the same time, inter-individual variability in response underscores the importance of considering genetic and epigenetic factors that may influence susceptibility to ethanol-induced injury and responsiveness to intervention [154].

Taken together, these findings support the concept that antioxidant supplementation represents a promising strategy for the prevention and early intervention of FASD. Future studies are needed to define optimal timing, dosing, and combinations of agents, with particular emphasis on safe implementation during critical windows of development.

### 5.2. Targeting Redox-Sensitive Signaling Pathways for FASD Prevention and Intervention

In addition to direct antioxidant supplementation, an emerging strategy for FASD prevention and intervention is the targeted modulation of redox-sensitive signaling pathways. These pathways, particularly Nrf2, NF-κB, and MAPK pathways, serve as key regulatory nodes that integrate oxidative stress with cellular responses during development. Pharmacologic or nutritional interventions that restore or rebalance these pathways have shown considerable promise in mitigating ethanol-induced developmental abnormalities in preclinical models (Table 2).

#### 5.2.1. Nrf2-Mediated Antioxidant Response as a Central Preventive Target

Among redox-responsive pathways, Nrf2 represents a principal endogenous defense mechanism against oxidative stress. Substantial experimental evidence demonstrates that enhancing Nrf2 activity can prevent ethanol-induced cellular injury and developmental defects. Genetic overexpression of Nrf2 in NCCs markedly attenuates ethanol-induced oxidative stress and reduces apoptosis, establishing a direct protective role for this pathway [48]. In vivo studies have shown that ethanol exposure activates an adaptive Nrf2 response in mouse embryos, which is insufficient to protect against ethanol-induced apoptosis; pharmacological activation of Nrf2 signaling by 3H-1,2-dithiole-3-thione (D3T) significantly mitigates embryotoxic outcomes [47]. Consistent with these findings, in cranial NCCs, tBHQ, a Keap1-modifying agent, enhances Nrf2 activity, reduces ROS accumulation, and prevents ethanol-induced apoptosis [46]. Similarly, D3T, which stabilizes Nrf2 protein, increases Nrf2 nuclear accumulation, enhances antioxidant response element (ARE)–driven transcription, and suppresses ethanol-induced oxidative damage in neuronal cells [155]. In addition, studies using dietary and naturally occurring Nrf2 activators further support the translational potential of this pathway. For example, sulforaphane has been shown to restore Nrf2 signaling, elevate antioxidant enzyme expression, and reduce ethanol-induced oxidative stress and apoptosis in NCCs [49]. Importantly, recent in vivo evidence demonstrates that sulforaphane attenuates ethanol-induced teratogenesis and vascular abnormalities in zebrafish embryos [156]. Likewise, resveratrol restores Nrf2 signaling and antioxidant capacity in the cerebellum, reducing ethanol-induced neurotoxicity [157]. Together, these findings identify Nrf2 activation as a mechanistically grounded approach to prevent FASD.

#### 5.2.2. Targeting NF-κB–Mediated Neuroinflammatory Signaling

Beyond antioxidant defense, modulation of redox-sensitive inflammatory signaling represents an important strategy for mitigating the neurodevelopmental consequences of prenatal and early postnatal ethanol exposure. NF-κB functions as a central transcriptional regulator linking oxidative stress to neuroinflammation, and its activation has been consistently associated with ethanol-induced brain injury. Studies have demonstrated that ethanol exposure during critical developmental windows induced a coordinated oxidative–inflammatory cascade, characterized by increased oxidative–nitrosative stress, elevated pro-inflammatory cytokines (e.g., TNF-α, IL-1β, and TGF-β1), and activation of downstream apoptotic signaling pathways, including NF-κB and caspase-3. These molecular alterations are accompanied by increased acetylcholinesterase activity and are closely associated with cognitive deficits in the cerebral cortex and hippocampus of ethanol-exposed offspring [158]. Pharmacological modulation of this pathway has shown significant protective potential [158,159,160,161]. Curcumin, a bioactive polyphenol with both antioxidant and anti-inflammatory properties, has been demonstrated to attenuate ethanol-induced neuroinflammatory signaling and its downstream consequences. In postnatally ethanol-exposed models, chronic curcumin treatment diminished ethanol-induced oxidative stress, inflammatory cytokine levels, and apoptotic signaling pathways, including NF-κB activation, and ameliorated behavioral impairments [158]. These findings indicate that suppression of the oxidative–inflammatory cascade can mitigate ethanol-induced neurotoxicity and associated functional deficits. Consistent with these observations, studies of prenatal and lactational ethanol exposure have shown that ethanol induces persistent neuroinflammatory changes, including increased expression of pro-inflammatory mediators, astrogliosis, and microglial activation, which are accompanied by anxiety-like behavior and memory impairments. Curcumin treatment significantly improved these behavioral outcomes and reduced neuroinflammatory responses [159]. Additionally, resveratrol downregulated MyD88 expression and reduced the phosphorylation of NF-κB, thereby disrupting the MyD88/NF-κB signaling cascade induced by ethanol, exerting a protective effect against ethanol-induced neuronal damage [160]. EGCG can also partly alleviate ethanol-induced endothelial injury by altering NF-κB translocation and activating the Nrf2 signaling pathway [161]. Collectively, these findings support the concept that NF-κB–mediated neuroinflammation represents a viable target for intervention.

#### 5.2.3. Modulation of MAPK Signaling Pathways

The mitogen-activated protein kinase (MAPK) signaling network, particularly the JNK and p38 MAPK pathways, represents an important downstream effector of ROS-mediated cellular stress and contributes to ethanol-induced cellular injury. Evidence indicates that ethanol-induced oxidative stress can activate MAPK signaling, thereby amplifying stress responses and promoting apoptosis. For example, studies in neuronal cell models have shown that ethanol-induced oxidative stress is mediated, at least in part, through activation of the p38 MAPK pathway, and pharmacological inhibition of p38 reduces ROS accumulation and attenuates cell death [64]. These findings highlight MAPK signaling as a key mediator of oxidative injury in neurons. Natural compounds capable of modulating MAPK signaling have also demonstrated protective effects. Quercetin, a dietary flavonoid, attenuates oxidative stress, lipid peroxidation, and mitochondrial dysfunction and suppresses JNK and p38 activation. In neuronal models, these effects are associated with reduced apoptosis and improvements in behavioral outcomes following ethanol exposure [162]. However, these findings are primarily derived from mature neuronal systems, and their relevance to early neurodevelopment remains to be investigated. Importantly, evidence from developmental models supports a direct role of MAPK signaling in ethanol-induced embryotoxicity. For instance, overexpression of microRNA-135a suppresses activation of the Siah1/p38/p53 signaling cascade, reducing apoptosis in neural crest cells and preventing craniofacial defects in zebrafish embryos exposed to ethanol [128]. These findings provide direct evidence that targeting MAPK-related signaling can attenuate ethanol-induced developmental abnormalities. Collectively, these studies indicate that MAPK signaling functions as a convergence point for ROS-mediated stress responses across both neuronal and developmental contexts. Targeting this pathway, either directly through kinase inhibition or indirectly via upstream regulatory mechanisms, may complement antioxidant and anti-inflammatory strategies to reduce ethanol-induced cellular injury and developmental defects.

### 5.3. Translational Challenges in Targeting Redox Dysregulation for FASD Prevention and Treatment

Substantial progress has been made in delineating how redox dysregulation contributes to the pathogenesis of FASD, and these insights have stimulated the development of antioxidant- and redox-sensitive pathways-targeted therapeutic strategies. Experimental models, particularly rodents and zebrafish, have been instrumental in demonstrating that prenatal alcohol exposure induces oxidative stress, neuroinflammation, and cellular injury across multiple tissues, including neural crest derivatives, the central nervous system, and cardiovascular structures [1,163]. These models have further shown that redox imbalance disrupts key developmental processes, including cell survival, proliferation, migration, and differentiation, thereby contributing to the structural and functional abnormalities characteristic of FASD [1,163]. In parallel, preclinical studies have provided proof-of-concept that targeting oxidative stress, either directly through antioxidants or indirectly via redox-sensitive signaling pathways, can mitigate ethanol-induced damage [152,164,165].

Despite these advances, significant challenges remain in translating preclinical findings into effective clinical interventions. One major limitation is the inherent difference between animal models and humans. Species-specific variations in ethanol metabolism, placental transport, brain maturation, and developmental timing can markedly influence both the severity of ethanol-induced injury and the efficacy of therapeutic interventions [163]. In addition, experimental paradigms often involve controlled exposure conditions that do not fully capture the heterogeneity of human alcohol consumption patterns, including differences in timing, dose, and duration of exposure. As a result, interventions that demonstrate efficacy in animal models may not directly translate to clinical settings. To address these limitations, there is increasing interest in developing human-relevant experimental systems. These include human neural crest cells derived from human embryonic stem cells [166,167], three-dimensional human brain organoids [168,169], and other advanced in vitro platforms that better recapitulate early human development and tissue-specific responses to ethanol. Such models provide opportunities to investigate human-specific mechanisms of redox dysregulation and to evaluate therapeutic candidates in a more physiologically relevant context.

Another major challenge is optimizing therapeutic strategies targeting redox imbalance. Current approaches encompass a broad range of interventions, including antioxidant supplementation (e.g., vitamins C and E, N-acetylcysteine), nutritional support (e.g., folate), and agents that modulate redox-sensitive signaling pathways [151,152,153,165]. It should be noted that modulation of pathways such as Nrf2 may be limited by the electrophilic nature of many activating compounds, which can lead to non-specific interactions, and by the strong dependence of their efficacy on the timing and cellular context of activation. Notably, therapeutic modulation of Nrf2 signaling is often constrained by the electrophilic properties of its activators, leading to potential non-specific effects, as well as by the context- and timing-dependent nature of its biological responses. In particular, targeting Nrf2 signaling is complicated by the electrophilic characteristics of many activators, which may result in non-specific protein modification, as well as by the context- and timing-dependent variability in therapeutic outcomes. While these interventions have shown promise in preclinical studies, their clinical application is complicated. The timing of intervention is particularly critical, as the vulnerability to ethanol-induced damage varies across developmental stages. Effective prevention may require early intervention, whereas postnatal treatments may primarily target secondary consequences rather than primary structural defects [152,153].

Moreover, although antioxidants can attenuate oxidative damage, indiscriminate suppression of ROS may have unintended consequences. Physiological levels of ROS are essential for normal cellular signaling, differentiation, and development, and excessive antioxidant supplementation could disrupt these processes [15,170]. This highlights the importance of restoring balanced redox homeostasis rather than attempting complete elimination of ROS. In addition, key pharmacological parameters, including bioavailability, placental transfer, metabolism, and potential interactions with maternal physiology or concurrent medications, remain incompletely understood for many candidate compounds. In addition, clinical evidence supporting redox-sensitive pathways-targeted interventions in FASD remains limited. Although some studies suggest potential benefits in improving neurodevelopmental outcomes or reducing structural abnormalities, the overall quality and consistency of clinical data are insufficient to establish standardized treatment protocols [151,153]. Furthermore, genetic and epigenetic variability among individuals may influence susceptibility to oxidative stress and responsiveness to intervention, underscoring the need for more personalized approaches [15].

Collectively, these challenges emphasize that, while targeting redox dysregulation represents a promising strategy for the prevention and treatment of FASD, substantial gaps remain between experimental findings and clinical application. Future research should prioritize the integration of human-relevant models, the identification of optimal therapeutic windows, and the design of well-controlled clinical trials. A more comprehensive understanding of redox biology in human development, combined with advances in precision medicine, will be essential for translating mechanistic insights into safe and effective interventions for FASD [15,153,165].

**Table 2:** Redox-modulating therapeutic agents for prevention and intervention in FASD.

Therapeutic Agents	Experimental Models	Key Findings/Outcomes	Refs
SOD	Mouse embryos	SOD diminished ethanol-induced superoxide production, lipid peroxidation, and cell death, and significantly reduced malformation in mouse embryos exposed to ethanol.	Kotch et al. [16]
SOD, CAT, α-tocopherol	Cranial neural crest cells	SOD, catalase, and α-tocopherol significantly reduced ethanol-induced NCC death.	Chen and Sulik [17]
EUK-134	In vivo Mouse model	EUK-134, a synthetic SOD/CAT mimetic, significantly reduced apoptosis and the incidence of ethanol-induced forelimb malformations in mouse embryos exposed to ethanol in vivo.	Chen et al. [130]
NAC	Mouse NCCs	NAC treatment diminished ethanol-induced cytotoxicity in mouse NCCs.	Chen and Sulik [21]
	In vivo mouse model	Dietary administration of NAC reduced ocular abnormalities in mouse embryos exposed to ethanol in vivo.	Parnell et al. [129]
Folic acid	Rats	Folic acid supplementation significantly attenuated ethanol-induced oxidative alterations in the liver and pancreas of offspring, indicating a protective role for folic acid in mitigating ethanol-mediated oxidative damage.	Cano et al. [138]
β-Carotene	Embryonic rat hippocampal culture	Supplementation with β-Carotene ameliorated ethanol-induced neuronal loss in embryonic rat hippocampal culture.	Mitchell et al. [140]
Vitamin E	Neonatal rats	Dietary supplementation of Vitamin E prevented ethanol-induced Purkinje cell loss.	Heaton et al. [141]
	Embryonic rat hippocampal culture	Vitamin E supplementation prevented ethanol-induced neuronal loss in the embryonic rat hippocampus.	Mitchell et al. [140]
EGCG	In vivo mouse model	EGCG treatment ameliorated ethanol-induced fetal growth restriction, attenuated disruptions in placental angiogenesis, and partially restored neurodevelopmental outcomes.	Almeida-Toledano et al. [142]
	In vivo mouse model	Postnatal EGCG therapy restored cardiac biomarkers and improved cardiac dysfunction in mice prenatally exposed to alcohol.	Andreu-Fernández et al. [143]
	Neonatal rat pups	EGCG significantly attenuated ethanol-induced cognitive impairment and biochemical/molecular alterations, including lower oxidative stress, inflammation, apoptotic markers, and improved antioxidant profiles.	Tiwari, Kuhad et al. [144]
	Rat fetal rhombencephalic neurons	EGCG prevented the ethanol-induced apoptosis in rat fetal rhombencephalic neurons.	Antonio and Druse [145]
	In vivo mouse model	Treatment with EGCG ameliorated ethanol-induced growth retardation, restored embryo size and neural marker gene expression, and inhibited increases in H_2_O_2_ and MDA.	Long et al. [146]
Resveratrol	Rat fetal rhombencephalic neurons	Resveratrol reduced ethanol-induced apoptosis in rat fetal rhombencephalic neurons.	Antonio and Druse [145]
	Postnatal rat pups	Resveratrol prevented ethanol-induced apoptosis in the cerebellar granule layer. This neuroprotection was associated with restoration of Nrf2 and downstream antioxidant targets in a rodent model of FASD.	Kumar et al. [157]
CBD	In vivo mouse model	CBD treatment normalized prenatal ethanol-induced emotional and cognitive disturbances and restored gene expression, cellular, and metabolomic alterations in the hippocampus and prefrontal cortex.	Gasparyan et al. [149]
	In vivo mouse model	CBD prevented cognitive impairments and neuroinflammation induced by early alcohol exposure in mice by restoring the ethanol-induced elevated TNF-α and IL-6 in the hippocampus.	Garcia-Baos et al. [150]
D3T	In vivo mouse model	Treatment with D3T enhanced Nrf2 activation, antioxidant gene/protein expression, and antioxidant enzyme activities. D3T also significantly reduced ethanol-induced ROS generation and apoptosis in mouse embryos.	Dong et al. [47]
	PC12 cells	D3T stabilized Nrf2 protein, promoted its nuclear accumulation, enhanced ARE-driven transcription, and suppressed ethanol-induced oxidative damage and cell death.	Dong et al. [155]
tBHQ	Primary cultured mouse cranial NCCs	tBHQ treatment significantly enhanced Nrf2 and antioxidant enzyme levels, reduced ROS generation, and attenuated ethanol-induced apoptosis in NCCs.	Yan et al. [46]
SFN	NCCs	SFN restored Nrf2 signaling, increased antioxidant enzyme expression, and reduced ethanol-induced oxidative stress and apoptosis.	Chen et al. [49]
	Zebrafish embryos	SFN attenuated ethanol-induced teratogenesis and vascular abnormalities in zebrafish embryos.	Wu et al. [156]
Curcumin	Rat fetal rhombencephalic neurons	Curcumin prevented the ethanol-induced apoptosis in rat fetal rhombencephalic neurons.	Antonio and Druse [145]
	Rat pups	Curcumin reduced oxidative stress markers, inflammatory cytokines, NF-κB activation, and apoptotic signaling in different brain regions and improved behavioral impairments in rat pups postnatally exposed to ethanol.	Tiwari and Chopra [158]
	In vivo mouse model	Curcumin improved ethanol-induced anxiety and memory deficits, and reduced neuroinflammatory responses.	Cantacorps et al. [159]

## 6. Conclusions and Future Directions

Prenatal alcohol exposure disrupts redox homeostasis during development and contributes to the pathogenesis of FASD. Accumulating evidence indicates that redox imbalance engages multiple interconnected signaling networks, linking oxidative stress to downstream effects, including impaired cell survival, altered differentiation, and neuroinflammation. These can interfere with coordinated developmental programs, ultimately leading to structural and functional abnormalities. In addition to mechanistic insights, growing experimental evidence supports the feasibility of preventive and intervention strategies targeting redox dysregulation. Approaches aimed at restoring redox balance, such as enhancing antioxidant capacity and modulating redox-sensitive signaling pathways, have demonstrated protective effects in multiple experimental models. Nutritional and pharmacological interventions, including antioxidant supplementation and bioactive compounds with antioxidant, anti-inflammatory, and signaling-modulatory properties, can attenuate ethanol-induced oxidative damage, reduce neuroinflammation, and improve cellular and behavioral outcomes. Notably, targeting redox-sensitive signaling pathways provides a more selective strategy than broadly suppressing ROS, allowing preservation of physiological redox signaling while mitigating pathological effects. These findings collectively support the concept that modulation of redox homeostasis represents a viable avenue for FASD prevention and intervention.

Future research should focus on the development and integration of human-relevant experimental models that better recapitulate the complexity of the effects of prenatal ethanol exposure on human embryonic development, which is critical for advancing translational applications. Combining human stem cell-derived models, organoid systems, and in vivo approaches will allow investigation of both cell-autonomous responses and multicellular interactions. Incorporating microphysiological systems or organ-on-chip platforms that model maternal–fetal interfaces may further improve the ability to recapitulate systemic aspects of prenatal ethanol exposure and enhance translational relevance. Another important direction is the identification of cell–type–specific and stage-specific redox regulatory networks. Identifying how distinct cell populations respond to redox perturbations will help define critical windows of susceptibility during which interventions may be most effective, providing a framework for precision prevention. Future studies should also aim to identify early biomarkers of redox imbalance and developmental risk. The discovery of circulating, imaging-based, or molecular markers that reflect early redox disruption could enable risk assessment and timely intervention. Linking such biomarkers to mechanistic pathways will be essential for translating experimental findings into clinical applications. Finally, translating mechanistic insights into effective prevention strategies will require optimization of intervention specificity, timing, and delivery. Approaches that selectively modulate redox-sensitive pathways, target specific sources of ROS, or enable tissue-specific delivery may provide greater efficacy while minimizing disruption of physiological signaling. Integration of experimental findings with clinical and epidemiological data will be essential for developing targeted, effective, and safe strategies to reduce the burden of FASD.

## Figures and Tables

**Table 1:** Redox-sensitive signaling pathways disrupted by prenatal ethanol exposure in FASD pathogenesis.

Signaling Pathways	Models	Key Findings/Outcomes	Refs
Nrf2/Keap1 signaling	Mouse embryos	Maternal ethanol treatment increased Nrf2 protein levels and Nrf2-ARE binding in mouse embryos, leading to a moderate upregulation of both mRNA and protein expression of Nrf2 downstream detoxifying and antioxidant genes. Induction of Nrf2 by D3T increased antioxidant response, decreased ROS, and prevented apoptosis in mouse embryos.	Dong et al., [47]
	NCCs	Exposure of NCCs to ethanol increased the protein expression of Nrf2 and catalytic activity of Nrf2 downstream antioxidants, SOD and catalase. tBHQ significantly increased Nrf2 and its downstream antioxidant expression, prevented oxidative stress and apoptosis in ethanol-exposed NCCs.	Yan et al., [46]
	NCCs	Overexpression of Nrf2 increased the protein expression and activities of Nrf2 downstream antioxidants in NCCs, significantly decreased ROS generation, and diminished apoptosis in ethanol-exposed NCCs.	Chen et al., [48]
	NCCs	Suppression of Nrf2 signaling in NCCs also significantly diminished SFN-mediated antioxidant response and the protective effects of SFN on ethanol-induced oxidative stress and apoptosis.	Chen et al., [49]
MAPK and PI3K/Akt signaling	NCCs	Ethanol upregulated Siah1 and triggered apoptosis in NCCs via p38 MAPK-mediated activation of the p53 signaling pathway.	Yuan et al., [50]
NF-κB signaling	*Xenopus* embryos	Ethanol exposure increased ROS production and activated NF-κB signaling in *Xenopus* embryos, contributing to ethanol-induced microencephaly and growth retardation.	Peng et al., [51]
Wnt/β-catenin signaling	Chick NCCs	Ethanol exposure resulted in sustained repression of β-catenin transcriptional activity, accompanied by downregulation of critical downstream effectors required for neural crest development and survival.	Flentke et al., [52]
	Zebrafish embryos	Calcium-dependent activation of CaMKII contributes to ethanol-induced apoptosis in zebrafish embryos.	Flentke et al., [53]
	Chick embryos	Ethanol exposure induced apoptosis in NCCs through an intracellular calcium transient that can activate CaMKII, which, in turn, destabilized transcriptionally active β-catenin.	Flentke et al., [54]
mTOR signaling	Rat	Chronic binge ethanol exposure during pregnancy altered mTORC1 signaling in the fetal hippocampus.	Lee et al., [55]
	Zebrafish embryos	Ethanol exposure in pdgfra mutant or heterozygous zebrafish embryos increased neural crest apoptosis and craniofacial defects, which could be mitigated by upregulating the mTOR pathway.	McCarthy et al., [57]
	Cranial NCC cell line	Ethanol disrupted ribosome biogenesis and triggered p53/MDM2-mediated apoptosis in NCCs by activating pAMPK, which suppresses TORC1/S6K-dependent ribosomal production through TSC2 and Raptor signaling.	Huang et al., [56]

## Data Availability

No new data were created or analyzed in this study. Data sharing is not applicable to this article.

## Abbreviations

The following abbreviations are used in this manuscript:

FASD	Fetal alcohol spectrum disorders
ROS	Reactive oxygen species
FAS	Fetal alcohol syndrome
ARND	Alcohol-related neurodevelopmental disorder
ARBD	Alcohol-related birth defects
PAE	Prenatal ethanol exposure
ND-PAE	Neurobehavioral disorder associated with prenatal alcohol exposure
NCCs	Neural crest cells
Nrf2/Keap1	Nuclear factor erythroid 2–related factor 2/Kelch-like ECH-associated protein 1
MAPK	Mitogen-activated protein kinase
PI3K/Akt	Phosphoinositide 3-kinase/Protein kinase B
NF-κB	Nuclear factor kappa-light-chain-enhancer of activated B cells
Wnt/β-catenin	Wingless/Integrated/β-catenin
mTOR	Mechanistic target of rapamycin
ADH	Alcohol dehydrogenase
MEOS	Microsomal ethanol-oxidizing system
CYP2E1	Cytochrome P450 2E1
NOX	NADPH oxidase
SOD	Superoxide dismutase
GPx	Glutathione peroxidase
CAT	Catalase
GSH	Glutathione
MDA	Malondialdehyde
NADH	Nicotinamide adenine dinucleotide
tBHQ	Tert-butylhydroquinone
SFN	Sulforaphane
CaMKII	Ca^2+^/calmodulin-dependent protein kinase II
MPT	Mitochondrial permeability transition
8-OHdG	8-hydroxy-2′-deoxyguanosine
JNK	C-Jun N-terminal kinase
ERK	Extracellular signal-regulated kinase
IκB	Inhibitor of κB
IKK	IκB kinase
EGCG	Epigallocatechin gallate
ER	Endoplasmic reticulum
CBD	Cannabidiol
D3T	3H-1,2-dithiole-3-thione
